# Diverse adolescents’ transcendent thinking predicts young adult psychosocial outcomes via brain network development

**DOI:** 10.1038/s41598-024-56800-0

**Published:** 2024-03-15

**Authors:** Rebecca J. M. Gotlieb, Xiao-Fei Yang, Mary Helen Immordino-Yang

**Affiliations:** 1 Center for Dyslexia, Diverse Learners, and Social Justice, School of Education and Information Studies, University of California Los Angeles, Los Angeles, USA; 2 Center for Affective Neuroscience, Development, Learning and Education; Brain and Creativity Institute; Rossier School of Education, University of Southern California, Los Angeles, CA USA; 3 Neuroscience Graduate Program; Psychology Department, University of Southern California, Los Angeles, CA USA

**Keywords:** Adolescent brain development, Longitudinal mixed methods, Social cognition, People of color, Intelligence, Morality, Human behaviour, Empathy, Social behaviour, Emotion, Intelligence, Motivation, Personality

## Abstract

Developmental scientists have long described mid-adolescents’ emerging capacities to make deep meaning about the social world and self, here called transcendent thinking, as a hallmark developmental stage. In this 5-years longitudinal study, sixty-five 14–18 years-old youths’ proclivities to grapple psychologically with the ethical, systems-level and personal implications of social stories, predicted future increases in the coordination of two key brain networks: the default-mode network, involved in reflective, autobiographical and free-form thinking, and the executive control network, involved in effortful, focused thinking; findings were independent of IQ, ethnicity, and socioeconomic background. This neural development predicted late-adolescent identity development, which predicted young-adult self-liking and relationship satisfaction, in a developmental cascade. The findings reveal a novel predictor of mid-adolescents’ neural development, and suggest the importance of attending to adolescents’ proclivities to engage agentically with complex perspectives and emotions on the social and personal relevance of issues, such as through civically minded educational approaches.

## Introduction

Adolescence is a period of marked cognitive, emotional and psychosocial growth^1^, as well as a sensitive period for neurological development^2^, the second such period after infancy. It is characterized by sensitivity to the social context and by the emergence of increasingly sophisticated abilities to interpret the social world and react with complex emotions to its happenings^3,4^. By middle adolescence, from approximately 14–18 years of age, youth develop the capacity for “transcendent” thinking. That is, mid-adolescents are disposed, and often motivated, to enrich their concrete, empathic, and context-specific interpretations with abstract, systems-level considerations that transcend the current situation^5–9^. They invoke broader perspectives on themselves, other people, and social systems, and draw on cultural values and associated emotions to infer social and ethical implications and build deeper understandings^10,11^. Moving into the later teenage years, transcendent thinking supports late-adolescents’ identity development, the process of building self-definitions rooted in reflections on experiences, hopes, relationships, values, and beliefs rather than on happenstance. As such, transcendent thinking may contribute to stronger identity achievement and less identity diffusion^12–14^. The identity development process can support a healthy transition to young adulthood^15–17^, in the early twenties, with emotionally fulfilling and stable relationships, a positive sense of self and life purpose^18^, and productive, ethical use of educational and work opportunities^19,20^. Especially among ethnically diverse youth, and youth from families of low-socioeconomic circumstances, transcendent social thinking and identity are important developmental assets, given the likelihood these youth will face complex circumstances and social challenges^11,21^.

Research in developmental science and education has long documented the academic and social benefits of supporting adolescents in building intellectual agency and developmentally appropriate capacities for thinking about complex social, civic, and academic disciplinary content^11,19,22,23^. Curiously, though, the affordances of mid-adolescents’ transcendent thinking for brain development have not been investigated. Neural maturation across mid-adolescence largely involves increasingly efficient communication among characteristic networks contributing to a range of developing psychological capacities^24–26^. This increasing efficiency is measurable in neural dynamics, i.e., in the real-time correlation between different neural networks’ activity fluctuations, even as individuals rest^24,27^. The change over time in the strength of these correlations, therefore, can be used as a metric of functional neural development. Across adolescence, there are considerable individual differences in these metrics. As these differences correlate with psychosocial functioning and mental health^25,27–30^, it is important to understand their origins^31^.

A previous study of ours revealed that mid-adolescents’ transcendent construals of social stories were associated with increased default mode network (DMN) activity, especially when the adolescent reported feeling strongly emotionally engaged with the story, and with decreased executive control network (ECN) activity^32^. In addition, the association between DMN activity and transcendence was strengthened by brief ECN activity early in the trial. These complex findings suggested that adolescents’ transcendent construals involve coordinated activity of the DMN, which supports internally generated reflections and prospections^33–35^, and the ECN, which supports goal-directed thinking and focused attention^36,37^. This interpretation is consistent with previous theoretical and empirical work documenting these networks’ coordinated involvement in many forms of creative, episodic, social-emotional and generative thinking^38–42^, and with research documenting links between maturation of these networks and social-emotional, cognitive and executive functioning and mental health^43–46^. Might the developmentally characteristic recruitment of transcendent thinking across mid-adolescence contribute to organizing connectivity between these two networks in ways that support long-term psychosocial growth and well-being?

Brain maturation during mid-adolescence is thought to reflect, in part, social and cognitive experiences, such as effects of socioeconomic and cultural backgrounds, education, and peer influences^4,47,48^. However, whereas exposure to social circumstances is an important source of experience, equally important may be adolescents’ growing propensities to grapple psychologically with what they witness, and make meaning^49^. Although IQ and demographic variables such as socioeconomic status (SES) have been associated with neurodevelopmental trajectories across childhood and early adolescence^46,50,51^, it is possible that their effect on longitudinal change across mid-adolescence may attenuate. Potentially, proclivities to expend effort on complex thinking may come to play a more prominent role.

To begin to investigate how mid-adolescents’ propensities for transcendent social-emotional thinking may predict subsequent brain development, with potential psychosocial implications in young adulthood, we launched a longitudinal 5-years study involving sixty-five 14–18 years-old youth of color from low-income urban communities. There is an urgent need in the psychological and brain sciences to study populations that have not traditionally been involved in research, i.e., non-White and low-to-mid SES samples, and in particular to focus on these populations’ normative development, rather than solely on deficits and risks^52^. At the time of recruitment, participants were healthy mid-adolescents from stable families, passing all classes at school, and not under disciplinary action. Our sample is diverse; participants speak English and were attending U.S. public high schools, but their parents had immigrated to the United States from thirteen different countries, primarily in East Asia and Latin America. Asian and Latinx youth are substantial and growing segments of the U.S. population. Our sample also is reflective of the variation in parental education levels and financial circumstances that exists within low-SES communities.

Participants explained their reactions to compelling mini documentaries in 2-h private interviews that were videotaped, transcribed, and coded for transcendent construals. Our interview provided participants with interesting, emotionally compelling true stories and a socially supportive situation conducive to reflection, and then allowed them to respond freely. Participants also underwent resting-state fMRI at the beginning of the study and after 2 years, to capture the longitudinal change in DMN and ECN internetwork connectivity. Identity development was surveyed after 1.5 more years, in late adolescence. In young adulthood, 5 years after initial data collection, when participants were in their early twenties, participants rated their satisfaction with self, relationships, and school to capture psychosocial well-being. We hypothesized that mid-adolescents’ construction of transcendent construals in the interview at the start of the study would predict increases in the DMN and ECN networks’ interconnectivity over the subsequent 2 years, regardless of the initial level of interconnectivity between these networks. We further hypothesized a sequenced developmental cascade in which the increases in these networks’ interconnectivity would in turn positively predict late-adolescent identity development, which would predict self and relationship satisfaction in young adulthood. To differentiate effects of transcendent thinking on brain network development from effects of age, family financial status and parents’ education levels (measures of SES), intelligence as measured by IQ, and other personal characteristics and demographic variables, we also measured, analyzed, and then controlled for these factors.

## Results

Every participant produced transcendent construals over the course of the interview at the start of the study (M = 25.14, *SD* = 14.00, range 2–64). Transcendent construal scores were not significantly related to IQ (*r*[59.9] = 0.23, *p* = 0.07, 95% CI [− 0.02, 0.45]), age (*r*[63] = 0.22, *p* = 0.08, 95% CI [− 0.02, 0.44]), or other demographic covariates (SES [family income/needs ratio and parents’ years of education], sex, ethnic background; all *p’s* > 0.44).

### Transcendent construal scores predict increases in internetwork connectivity over time

Consistent with our hypothesis, adolescents’ transcendent construal scores predicted the increase in connectivity between the DMN and the left ECN components across the 2-years interval following the interview, controlling for differences in head motion between the two neuroimaging data collections and time between data collections (*b* = 0.007, *SE* = 0.003,* t*[55.9] = 2.70, *p* = 0.009; bootstrapped 95% CI [0.002, 0.012]). Results hold in a model additionally controlling for age, sex, IQ, SES and starting level of connectivity between these components (*b* = 0.005, *SE* = 0.002, *t*[49.2] = 2.26, *p* = 0.03; bootstrapped 95% CI [0.0005, 0.0094]). The effect of transcendent construals on growth in internetwork connectivity was not significantly moderated by age, sex, IQ or SES (all *p’s* > 0.44).

#### Ethnic background

Given current discussions about the generalizability of psychological effects across ethnically diverse samples^52,53^, the effect of transcendent construals on longitudinal change in DMN and left ECN interconnectivity was examined in a model with participants divided into two broad ethnic groups: East-Asian descent (*n* = 29) and Latinx/Afro-Latinx descent (*n* = 36). The effect of transcendent construals holds (*p* = 0.008). Ethnic group did not moderate the effect of transcendent construals on change in internetwork connectivity (*p* = 0.71).

#### Laterality

Results were lateralized to the left ECN. Change in connectivity between the DMN and right ECN components across the 2-years interval was not predicted by transcendent construal scores, controlling for difference in head motion between the two data collections and time between data collections, *p* = 0.82.

### The sequenced developmental cascade from transcendent construals in mid-adolescence to young adult life satisfaction

Psychosocial outcomes varied across participants (identity development, a composite measure of identity achievement and diffusion: *M* = 3.69 out of 5, *SE* = 0.09; life satisfaction, a composite measure of satisfaction with self, various social relationships, and school [for the 49 still attending school]: *M* z-score = 0.05, *SE* = 0.11).

**Figure 1:**
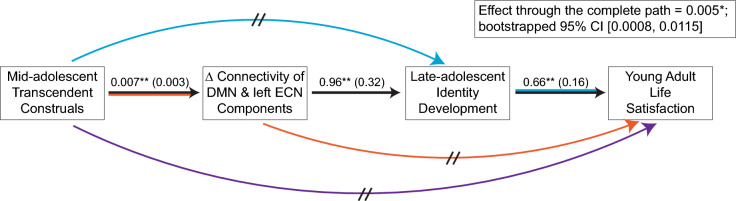
The longitudinal path from transcendent construals in mid-adolescence to life satisfaction in young adulthood, through 2-year change in resting-state internetwork connectivity (Δ Connectivity), and identity development in late adolescence. Consistent with a developmental cascade, only the complete path (black arrows) is significant; see inset. Alternative paths that omit either (blue arrows, red arrows) or both (purple arrow) of the intermediate measures are not significant. Regression coefficients are depicted for the complete path, with standard errors in parentheses. DMN, default mode network; ECN, executive control network; ** *p* < 0.01, *p < 0.05, slashes signify a non-significant relationship; CI, confidence interval.

Developmental measures were chronologically ordered, and a series of regression models revealed significant effects from one to the next. A path analysis then revealed that the complete path is significant, while alternative paths that omit either or both of the intermediate measures are not. The findings together suggest a developmental cascade; see Fig. 1. All models control for differences in head motion between the two scans and time between scans.

## Discussion

For at least a century, developmental theorists have described adolescents’ emerging abilities for transcendent social thinking, known also as abstract thinking, as a hallmark developmental stage^6,8,12,54^. Here, we demonstrate that adolescents’ proclivity to engage with such thinking predicts key, large-scale brain networks’ increasing interconnectivity over time and that this neural development is, in turn, associated with personal and social well-being in young adulthood. Importantly, in our socioeconomically and ethnically diverse urban sample, IQ and demographics did not explain the findings.

We focused on the default mode and executive control networks in particular because we had previously demonstrated that mid-adolescents’ transcendent construals were associated with coordinated activity in these networks during a functional task^32^. Extensive research suggests that these networks support reflective, autobiographical and free-form thinking, and effortful, focused thinking, respectively^34–36^. These networks’ coordinated activity is associated with many forms of generative and social-emotional processing^38–41^, and with mental health, including among adolescents^45^. Our study associates the positive developmental coordination of these networks with mid-adolescents’ emerging dispositions to construct transcendent meaning, inferring the broader, systems-level, ethical and civic implications, and emotionally poignant values and personal lessons, that extend beyond the immediate social situation.

Our study utilized an open-ended interview approach that aimed to capture the patterns of thinking participants spontaneously employed as they reacted to compelling, true social stories. There is a long history in constructivist developmental science of examining individuals’ processes of thinking independent of the specific content; this approach allows researchers to capture developmentally characteristic ways of thinking^6,8,12,55^. Building from this tradition, we analyzed not “what” youth were thinking, whether they agreed with the protagonist’s choices or the values the protagonist appeared to endorse, but “how” they were thinking, whether they showed evidence of considering the broader implications of the story for themselves or the world. Similarly, identity development measures captured the degree to which a participant had deliberated on their values and views, without regard to what they had decided. Notably, every participant produced transcendent construals during the interview, and therefore was capable of transcendent thinking. Given this finding, our method arguably assesses adolescents’ developing agentic *dispositions* toward transcendent thinking—how much they spontaneously invoke this cognitive-affective process, given a situation or domain that invites it. Ultimately, transcendent thinking may be to the adolescent mind and brain what exercise is to the body: most people can exercise, but only those who do will reap the benefits.

Our findings also speak to the value of utilizing in-depth qualitative research in developmental cognitive neuroscience to begin to understand meaningful sources of individual variation in brain development, and meaningful implications of this variation for outcomes. Our modest sample size was chosen to make possible a natural-feeling experimental protocol that encourages participants to engage genuinely with the true stories we shared, and to feel comfortable taking the time to figure out and explain their responses. Although future research might possibly design a more efficient means of uncovering adolescents’ psychological propensities, the ecological validity intended in our approach allowed us to uncover a critical interindividual difference in intraindividual change^56^. The focus on large-scale neural networks allowed us to align a broad psychological capacity with an equivalently broad neurodevelopmental pattern, facilitating interdisciplinary interpretation of the findings^57^. The data provide a developmental cascade that is consistent with theoretical accounts of self-construction within the dynamic developmental system^8^, i.e., with the notion that youth actively contribute to their own development^23^. Our previous reports that, at the start of the study, participants’ patterns of construals were associated with real-world social and cognitive functioning^7^, and with trial-by-trial activity in the brain^32^, strengthen this interpretation.

Related, the findings speak to the power of longitudinal designs for understanding the ontogeny of interindividual variation in outcomes, and possible targets for intervention. By focusing on participants’ neural connectivity *change scores*, embedded in a longitudinal path of characteristic psychosocial developmental achievements—we are able to learn about a trajectory of neural change that is associated with later psychosocial health, and about a psychological precursor of that change. The change-score logic is similar to that of pediatricians who record infants’ eating and weight across time not because they are primarily interested in how a baby’s weight compares to that of their same-age peers (assuming the baby is within a normal weight range), but because they aim to describe the infant’s rate of weight gain relative to that of their peers. It is this rate that is associated with health outcomes. Pediatricians would also advise parents about the modifiable factors that effect the rate of weight gain, such as feeding. Analogously, our study found that neural change over time was associated with later psychosocial development indicative of young adult psychosocial health, and that transcendent thinking predicts this neural change. This finding underscores the importance of the developmental process cascading from transcendent thinking, and points the way for future studies that would strategically assess whether and when interventions could be effective.

Future research should also focus on the developmental origins of individual variation in mid-adolescents’ transcendent thinking, especially as transcendent thinking is context dependent and appears to be malleable over time^9^. Given adolescents’ expanding social sphere, our data suggest the possibility that adolescents’ emerging disposition for thinking transcendently about what they encounter may itself be a source of variation in how the brain develops over time, akin to the association between eating well and growing well as an infant. Schooling, community-based programming, and parenting that support adolescents’ capacities to generate culturally relevant meaning of their social world, and to build identity, produce lasting benefits^23^. These can include improved academic performance and persistence in school^19,21,58,59^, increased sense of life purpose^9^, and improved biological markers of health^60^.

Further, future studies linking developmental origins of transcendent thinking to earlier brain development and social circumstances might possibly contribute to sorting out aspects of the trajectory of neural development across adolescence. In general, more protracted periods of structural development, and higher levels of internetwork connectivity and neural network segregation, have been associated with beneficial life circumstances and psychological outcomes, as indexed mainly by SES and IQ^51,61^. However, while neural network integration generally increases across this age^24^, research on the relations between SES and functional neural development of resting state network connectivity in adolescence has produced conflicting findings^51,62,63^. The relative lack of clarity on these topics may reflect in part the complex dynamics of thought and emotion emerging at this age, and in part the limitations of the predictive constructs. In particular, measures of SES and IQ are coming increasingly under scrutiny for their potential cultural biases and, in the case of SES, for their inability to capture critical variation in youths’ family and community-level social supports and cultural assets that may facilitate patterns of thinking like those we capture here^64–66^. Future research should investigate the value of more naturalistic, ecological methods for studying the psychological correlates and predictors of brain development, and especially of methods that capture youths’ strengths and not simply their environmental liabilities.

From a more applied perspective, future research should test the causal nature of the relationship between transcendent thinking and future neural and psychosocial development, and the ways that exposure to educational and clinical practices designed to support increases in transcendent thinking may contribute to future growth. For example, research on the possible neurobiological effects of civically-oriented community schooling^19^ and restorative justice approaches^59,67^ could lead to insights for developmental science while contributing useful evidence for education, mental health, and juvenile justice reform^22,57^. Examining the ways that adolescents’ transcendent thinking can be leveraged for both prosocial and antisocial aims, toward healthy and unhealthy outcomes, is another important future direction. Our assessment of transcendent construals was value neutral; we did not judge the prosociality or normativity of a participant’s response. (That said, we note that our participants were screened for psychiatric diagnoses and serious disciplinary infractions, and their transcendent construals were overwhelmingly prosocial.)

We hope that our findings provide a source of developmental hypotheses for larger, longer-term longitudinal studies with the power to examine more nuanced neurological and psychological effects across a wide range of participants^68^, and normative cross-sectional ranges of neural connectivity and transcendent thinking at different ages. Ongoing research is identifying subtle patterns of longitudinal integration and differentiation in neural network functioning^27,30,47,69^. Many of the regions changing with development contribute to the cognitive and affective processing undergirding transcendent thinking and its components, such as emotional feelings, autobiographical memory, motivation and reward processing, and self-processing^28,48,70^. Youth will almost certainly vary on the kind of transcendent thinking processes they preferentially invoke, e.g., self-relevant versus systems-oriented, and therefore on their relative reliance on the different affective and cognitive component processes. Larger studies would be positioned to explore developmental effects with greater granularity, in youth exposed to a range of social, cultural and educational contexts, and to probe connections to various domains of information processing not explicitly social^57^. Larger studies would also be positioned to investigate the possibility of contextual moderation effects that were not detectable in our moderately-sized sample.

It is hard to imagine a human context in which the capacity to engage in transcendent thinking would not confer benefits, assuming that we collectively aim for wellness and an ethical society capable of interrogating structures and systems, and of innovation. By middle adolescence, youth are oriented to, and even agentically dedicated to, engaging in such thinking. As a result, they can count among society’s most idealistic and committed citizens. The disposition to build complex, values-based inferences about the personal, social and ethical implications of the situations we encounter, and to become curious about the reasoning behind complex societal systems, is uniquely human. The proclivity to think about issues and beliefs that transcend proximal goals and the current context is the basis for adult-like moral values, identity development, civic participation and a sense of purpose^18^. Our study suggests that as mid-adolescents engage in transcendent thinking, trying on their newly expanding capacities for making meaning, they coordinate neural networks involved in effortful thinking and internal reflection. This spontaneous, active coordination across development may contribute to the growth of both their brains and their minds, lifting them over the threshold to productive young adulthood.

## Materials and methods

These data were collected as part of a larger project, for which participants also completed psychosocial activities, psychophysiological recordings and neuroimaging unrelated to the present study (e.g., interviews about school; studies of heart-rate variability; diffusion tensor imaging; see https://osf.io/gqs34 for more information). Methods for analyzing participants’ interview responses are described extensively in Gotlieb et al.^7^. The current study is the first to report longitudinal findings, and the first to analyze resting-state network connectivity and psychosocial outcome data. All study activities were approved by and carried out in accordance with the policies of the Institutional Review Board of the University of Southern California (UP-12-00206). All parents/legal guardians and participants gave written informed consent or assent as appropriate, and all participants were compensated for their time.

### Participants

65 youth (36 female) were recruited from public high schools in low-SES neighborhoods in Los Angeles. All participants were right-handed, aged between 14 and 18 years at the time of the initial data collection, fluent in English and passing all classes in school; none were under school disciplinary action. None had a history of drug/alcohol use or neurological/psychiatric issues. Participant characteristics are as reported by participants and as confirmed by parents/legal guardians and teachers (to the best of their knowledge). Characteristics of the sample were as follows: 51 participants reported receiving free or reduced-price lunch at school (indicating low income/needs ratio for the family^71^, and therefore low-SES). Parents’ education, an additional factor associated with SES^50^, ranged from 8 to 18 years (*M* = 12.4 years, *SD* = 3.8). 34 participants identified as Latinx, 29 as East Asian, and 2 as Afro-Latino; participants’ parents were born in 13 countries. Participants ranged in age from 14 to 18 (*M* = 15.77 years, *SD* = 1.05) at the start of the study. IQ scores ranged from 79 to 131 (*M* = 103.6, *SE* = 1.52; see below for relevant methodological details).

### Procedure

#### Interview

Adapting a previously established protocol (see Immordino-Yang et al.^72^), participants reacted to 40 true, compelling stories about living, non-famous adolescents from around the world in a range of circumstances, during a 2-h private video-taped interview. The story corpus was previously piloted to be interesting and to elicit mixes of positively and negatively valenced emotions. The experimenter shared each story using a previously memorized script, and then played an accompanying documentary-style video of approximately 1 min in length depicting footage of the real-life protagonist (not an actor), using PowerPoint (Microsoft Office) displayed on a Lenovo laptop with a 17-inch screen. After showing each video, the experimenter asked, “how does this story make you feel?” The experimenter then looked down and transcribed as much as possible of the participant’s verbatim responses by hand-written notes. Participants were told that notetaking was conducted in case the video camera failed. Actually, these notes also served to standardize the experimenter’s behavior, so that the participant could respond freely. Participants were encouraged to be as candid as possible.

#### Neuroimaging

Following the interview and a short break, participants underwent a 7-min resting-state BOLD fMRI scan with simultaneous pulse monitoring. Participants were instructed to think about whatever they would like, to stay as still as possible, and to stay awake. An image of a nature scene without people or animals was displayed continuously. Participants returned to the lab to repeat the scan approximately 2 years later (*M time between scans* = 2.10 years, *SD* = 0.21, range = 1.94–3.28 years). The protocol was timed so that scans would occur in the middle of the day.

##### MRI data acquisition

BOLD fMRI scanning at initial data collection was conducted with a 3 Tesla Siemens Trio scanner and a 12-channel matrix head coil. Functional scans were acquired using a T2^∗^-weighted echo-planar imaging (EPI) sequence (TR = 2 s, TE = 25 ms, flip angle = 90°, acquisition matrix: 64 × 64, FOV = 192 mm) with a voxel resolution of 3 × 3 × 3 mm^3^. Forty-one continuous transverse slices were acquired in interleaved order to cover the whole brain. A total of 210 volumes were acquired during the 7-min resting state scan. Anatomical images were acquired using a magnetization-prepared rapid acquisition gradient echo (MPRAGE) sequence (TI = 800 ms, TR = 2530 ms, TE = 3.09 ms, flip angle = 10º°, isotropic voxel resolution of 1 mm^3^; acquisition dimensions: 256 × 256 × 176). At the second data collection, a 3 Tesla Siemens Prisma scanner with 20-channel matrix head coils was used due to a system upgrade at the scanning facility. Scanning parameters remained the same. (N.B.: the analysis focuses on interindividual effects, which would be independent from any effects of the scanner upgrade.)

##### Pulse oximetry data acquisition

Pulse oximetry was acquired using an MRI-compatible oximeter (Nonin Medical Inc, 8600FO MRI, Plymouth, MN, USA) placed over the middle finger of the participant’s left hand; data were output to a BIOPAC MP150 system and recorded using the BIOPAC Acqknowledge software (version 4.1; BIOPAC Systems Inc., Goleta, CA, USA).

#### IQ testing

After the second fMRI scan, a trained experimenter individually administered the vocabulary and matrix reasoning subtests of the Wechsler Abbreviated Scale of Intelligence, second edition^73^ in a private room. Subtests were scored, age normed and summed to produce total IQ scores. One participant completed only the vocabulary subtest due to time constraints; we imputed overall IQ from this score.

#### Psychosocial survey measures

Approximately 3 years, 4 months after the initial data collection, participants completed a modified and abridged version of the Objective Measure of Ego Identity Status instrument^74,75^ via an online survey that they received via electronic communication (i.e., email, text message, and/or social media direct message). Three questions measured identity achievement (“I have gone through a period of serious questions about my values”; “I have developed my own viewpoint on what is best for me”; “I engage in self-exploration and discussions with others to figure out my views on life”). Two questions measured identity diffusion (“I just hang with the crowd”; “I sometimes join activities when asked, but I rarely try anything on my own”). All used a 5-point Likert scale, from “not at all true” to “completely true.” A composite identity development score was calculated from the average of responses on the Identity achievement questions and identity diffusion questions (reverse scored).

Approximately 5 years after initial data collection, participants completed an online survey of life satisfaction to capture well-being. Participants reported their satisfaction with their social relationships (as many as pertain; 7-point Likert scale; one question each for parents, siblings, friends, teachers/supervisors, romantic partner, children), with themselves (continuous sliding scale; “how satisfied are you with whom you have become?”), and with school (only if relevant; continuous sliding scale; “how much do you like school?”). A composite life-satisfaction score was calculated from the average of *z*-scores for: satisfaction with self; satisfaction with school (if relevant); and satisfaction with social relationships (calculated as an average of reported values). All survey instruments were administered using Qualtrics software (Provo, UT).

### Analysis

#### Transcendent construals

For our previous study, videotaped interviews had been transcribed and verified. Each participant response had then been blind coded and reliability coded for transcendent construals (See Gotlieb et al.^7,32^). Building from our previous work, transcendent construals were defined as utterances reflecting:

(i) systems-level analyses or moral judgements, or curiosities about how and why systems work as they do, e.g.,

“I also find it unfair that the people get undocumented. It’s kind of weird how it’s like a label how like just ‘cause you are from some other place, um, you can’t do certain things in another place. It’s like a question. It’s like something I’ve always wondered…”;

(ii) discussions of broad implications, morals and moral emotions, perspectives, personal lessons or values derived from the story, e.g.,

“I think back to the idea that because children are the future […] we have to be able to inspire people who are growing and have the potential to improve the societies”;

“it makes me happy for humanity”;

or (iii) analyses of the protagonist’s qualities of character, mind, or perspective, e.g.,

“[she is] thinking, ‘oh, you’re not alone. You have others who are dependent on you’.”

Importantly, it was not relevant whether the participant endorsed a value or lesson or agreed with the protagonist, e.g.,

“I wouldn’t react that way. I’d just be really mad at the kid instead of, you know, selfless like that and trying to help him. Like I wouldn’t be able to put myself in someone’s shoes like that like he did.”

Construals not considered transcendent pertain mainly to discussions of the protagonist’s immediate situation, e.g., “I’m glad it all worked out,” or evaluating the protagonist’s decisions or actions, e.g., “I feel like they should have planned it more”; or to the empathic emotions of the participant, e.g., “I feel really sad for her, and like, second-hand embarrassment”. Unlike transcendent construals, these examples involve reactive, concrete and context dependent interpretations.

Participants received a score of 1 for each transcendent construal. Scores across all trials were summed to produce a total score for each participant.

#### Neural data processing

##### fMRI data pre-processing

MRI data underwent standard preprocessing using SPM12 (v.7771) implemented in MATLAB 2015b (Wellcome Department of Cognitive Neurology, London, UK; MathWorks, Inc., Natick, MA, USA). Functional images were slice timing and motion corrected, and co-registered to the anatomical image. Anatomical images were normalized to the Montreal Neurological Institute space using the segmentation procedure. The resulting normalization transformation was applied to the functional images. Co-registered and normalized images were visually inspected for each participant to ensure quality, and all were satisfactory. The functional images were resampled into an isotropic voxel resolution of 2 × 2 × 2 mm^3^ and smoothed using an 8 mm full width at half maximum Gaussian kernel. To quantify and evaluate head motion, framewise displacement (FD^76^) was calculated. Across all scans, the number of volumes across the scan with FD over 1 mm ranged from 0 to 63 out of 210 (M = 4.0, SD = 8.6); the average FD across the resting state scan ranged from 0.06 to 0.92 mm (M = 0.19, SD = 0.13). No data were discarded due to head motion, though special care was taken to ensure that network identification and the associated connectivity measures are not biased by motion, either of the head or due to cardiac pulsation; see SI Sect. 1.

##### Neural network identification

**Figure 2:**
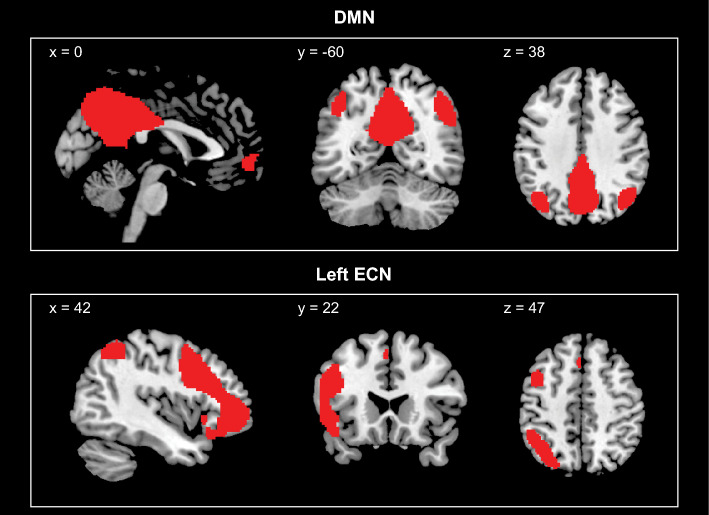
Depiction of coronal, sagittal, and axial views of the group-level default mode network (DMN; top) and left executive control network (left ECN; bottom) maps, derived from a 20-component group independent component analysis of the resting-state data (concatenating data from all participants from the two data collections), transformed into *z*-score maps and thresholded at *z* = 2.

Resting-state data were run through a 20-component group-level spatial independent component analysis^77^ using the Infomax algorithm (as implemented in the GIFT toolbox version 4.0b, http://mialab.mrn.org/software/gift/index.html). Group-level ICA was run without additional denoising because this approach has been shown to identify the networks of interest most accurately^78^. The 20-component model was chosen because it has been shown to capture large-scale networks, including those of interest here^79,80^. The first 5 TRs of the resting state scan were excluded to allow for signal stabilization. Consistent with standard practice, the algorithm was run 20 times with different initial values to evaluate the reliability of results using the ICASSO^81^ function in the GIFT toolbox. All components were highly reliable, with stability indices greater than 0.96^81^. Group-level components were then back-reconstructed to create individual-level component spatial maps and corresponding component time-courses for the initial and second data collections. Component spatial maps were visually inspected; components containing the left ECN, right ECN, and the DMN, were identified (see Fig. 2 and SI Sect. 2). Cross-correlation values between the identified network component maps and the resting state templates from Smith et al. (2009)^80^ were calculated using the fslcc function from the FMRIB Software Library (Version 6.0.6.5). Cross-correlation values for the group-level components are DMN: 0.77; left ECN: 0.74; right ECN: 0.72, considered high^82^.

##### Network functional connectivity

Network functional connectivity was calculated using the Mancovan toolbox^83^ implemented in GIFT. Default corrections and, for additional confidence, motion corrections, were applied for the ECN and DMN component time courses. This step included linear, quadratic, and cubic detrending; de-spiking; low-pass filtering with a high frequency cutoff of 0.15 Hz; and, to remove any residual influence of head motion, regressing out twenty-four expanded motion parameters^84^. Pairwise between-network correlation coefficients were calculated using corrected time courses across the entire scan (205 TRs) and then Fisher z-transformed to capture the strength of functional connectivity between networks for the initial data collection and second data collection separately for each individual. To capture longitudinal change, the difference in between-network correlation coefficients for the initial and second data collections for each participant was calculated.

#### Statistical analysis

Statistical analyses were carried out using RStudio (Version 2023.06.0 + 421, Posit Software, PBC) and R (Version 4.3.1). All reported statistical tests are 2-tailed. Data and R scripts are available at: https://osf.io/6cejy.

##### Missing data and multiple imputation

All participants had complete data from the initial collection. Missing neuroimaging data at the second data collection were due to unexplained extensive signal loss (1 participant), acquiring dental braces or metal implants (5 participants), or moving away (4 participants). Only two participants attrited after completing the initial data collections; all other missing data were partial. The percentage of missing values across the variables of interest varied between 0 and 15%. Given the reasons for the missing data are known and unlikely to be related to the measures of interest, the data were assumed to be missing at random and appropriate for multiple imputation^85^. (See also sensitivity analyses, described below.)

Missing data were imputed under fully conditional specification using predictive mean matching with 10 maximum iterations, as implemented in the Multivariate Imputation by Chained Equations (“mice”) package^86^ (Version 3.16). All existing data, including measures of interest and covariates, were used to conduct imputation. To stabilize results, 100 imputed datasets were produced. All difference scores were calculated after imputation.

##### Calculating bootstrapped confidence intervals

5000 bootstrapped samples were generated using the Bootstrap Functions (“boot”) package^87^ (Version 1.3–28.1) from each of the 100 imputed datasets, following Wu & Jia’s method^88^ for combining bootstrapping with multiple imputation. Effects of interest were estimated using each imputed/bootstrapped sample. The resulting 100 sets of 5000 parameter estimates were combined into one distribution for each effect of interest, which was used to derive the mean and a 95% confidence interval using the percentile method^89^.

##### Testing hypothesis 1

The effect of transcendent construals on longitudinal changes in between-network functional connectivity was examined using a series of fixed effect linear regression models to examine the hypothesized effect and then to confirm the effect when including additional relevant control and moderation terms. All analyses control for differences in average FD (head motion) between the two scans and time between scans. Models were run based on each imputed dataset. Two methods for significance testing were utilized to assure robustness. In the first, statistics of interest were pooled using Rubin’s rules^85^ for averaging regression coefficients, combining associated variances, and calculating degrees of freedom and an associated *p* value (as implemented in the “mice” package^86^). In the second, all models were estimated again using each of the imputed/bootstrapped samples to provide a confidence interval as described above.

##### Testing hypothesis 2

The effect of transcendent construals on life satisfaction through internetwork connectivity change and identity development, and through alternative paths that omit either or both of these intermediate measures, was tested. To do this, relevant measures were chronologically ordered to construct a developmental path model. Then, following the method described in Hayes^90^, for each of the imputed/bootstrapped datasets, three regression models were estimated using ordinary least squares, structured such that each subsequent measure in the path is predicted by the previous measures. These regression models controlled for differences in average FD (head motion) between the two scans and time between scans. Next, effects through the four possible paths (represented by the colors in Fig. 1) were calculated as the product of regression coefficients along the path. Significance testing was carried out based on the distribution of resulting parameter estimates, as described above.

#### Sensitivity analyses

Several analyses were run to give assurance that the findings are not biased by methodological decisions. To confirm that the findings hold in the sample without imputing missing data, the analyses were run with only complete cases. All findings hold; see SI Sect. 3.

To address the possibility that the missing psychosocial data could have violated the missing-at-random assumption, we used the post-processing procedure from the “mice” package^86^ to systematically vary imputed datapoints from what they would be under the missing-at-random assumption, to values corresponding to plus/minus 20% of the range of existing data. All findings hold.

### Supplementary Information

Supplementary Information.

## Data Availability

The data that support the findings of this study are available at https://osf.io/6cejy.

## Code availability

The R scripts for statistical analyses presented in the paper are available at https://osf.io/6cejy.
