# Supplementary material for: Diverse adolescents’ transcendent thinking predicts young adult psychosocial outcomes via brain network development
Source: Sci Rep. 2024 Mar 15;14:6254. doi: 10.1038/s41598-024-56800-0 (PMC10943076; doi:10.1038/s41598-024-56800-0)
Supplement: Supplementary file 1 — Supplementary Information. [file 41598_2024_56800_MOESM1_ESM.pdf]

Diverse Adolescents' Transcendent Thinking Predicts Young Adult Psychosocial Outcomes via Brain Network Development

Rebecca J.M. Gotlieb[a†], Xiao-Fei Yang[b†], and Mary Helen Immordino-Yang[b,c*]

[a] Center for Dyslexia, Diverse Learners, and Social Justice, School of Education and Information Studies, University of California Los Angeles, USA

[b] Center for Affective Neuroscience, Development, Learning and Education; Brain and Creativity Institute; Rossier School of Education, University of Southern California, Los Angeles, California, USA

[c] Psychology Department; Neuroscience Graduate Program, University of Southern California, Los Angeles, California, USA

*Corresponding Author: Mary Helen Immordino-Yang

E-mail: immordin@usc.edu

†Rebecca Gotlieb and Xiao-Fei Yang contributed equally to the study.

Author contributions: RG, X-FY, and MHI-Y designed research, performed research, analyzed the data and wrote the paper.

Competing interest statement: The authors declare no competing interests.

Keywords: Adolescent Brain Development; Longitudinal Mixed Methods; Social Cognition; People of Color; Intelligence

Supplementary Information

1. Head Motion and Cardiac Pulsation

The following analyses were conducted to confirm that our findings could not be attributed to head motion or cardiac pulsation:

A. Relationships between head motion measures (number of volumes with framewise displacement [FD] exceeding 1mm; average FD) and transcendent construal scores were examined in the data collected at each timepoint separately, and on the intraindividual change in motion between timepoints; all p's > 0.42.

B. ICA analysis was repeated using the portion of the sample ($n = 27$) with lowest head motion, as a stringent data scrubbing procedure. Across participants and across the two data collections: the number of volumes across the scan with FD over 1 mm ranged from 0 to 5 out of 210 ($M = 0.6$, $SD = 1.3$); the average FD across the resting state scan ranged from 0.07 to 0.24 mm ($M = 0.13$, $SD = 0.04$). The relationship between transcendent construal scores and change in network connectivity between DMN and left ECN holds in this portion of the sample ($b = 0.01$, $SE = 0.004$, $t[23] = 3.31$, $p = 0.003$), controlling for differences in head motion between the two neuroimaging data collections and time between data collections. Additionally controlling for age, sex, IQ, SES, and starting level of connectivity between these components (i.e., all covariates), the relationship between transcendent construal scores and change in network connectivity between DMN and left ECN remains significant, $b = 0.008$, $SE = 0.003$, $t[17] = 2.69$, $p = 0.02$.

C. As a final confirmatory analysis, we additionally accounted for the variability related to cardiac pulsation. Pulse waveform peaks were identified using the Acqknowledge software (version 4.1; BIOPAC Systems Inc., Goleta, CA, USA) and visually inspected. Misidentified peaks were manually corrected. The resulting pulse peak timing information was processed using the Translational Algorithms for Psychiatry-Advancing Science (TAPAS) toolbox[1] (Version 6.0.1) to calculate cardiac RETROICOR regressors[2]. This method models cardiac-related physiological noise as a low-order Fourier expansion (model order = 3, for 6 terms) of the cardiac phases.

We recalculated the internetwork connectivity scores accounting for the 6 cardiac regressors and the 24 terms associated with head motion. Using these new scores, all models in the manuscript hold: Adolescents' transcendent construal scores predicted the increase in connectivity between the DMN and the left ECN components across the two-year interval following the interview, controlling for differences in head motion between the two neuroimaging data collections and time between data collections ($b = 0.007$, $SE = 0.003$, $t[55.2] = 2.75$, $p = 0.008$; bootstrapped 95% CI [0.003, 0.012]). For the developmental cascade, the complete path is significant (effect through the complete path = 0.005, bootstrapped 95% CI [0.0006, 0.0110]), while alternative paths that omit either or both of the intermediate measures are not.

2. Evaluating Network Component Maps.

The IC's identified in our analysis are very similar to the templates derived from large-scale 20-component group ICA studies (e.g., Smith et al., 2009)[3]. A visual inspection of the properties of the 20 components stably identified by our analysis reveals that component 11 captures the DMN; component 12 captures the left ECN; component 15 captures the right ECN;

component 6 captures head motion; component 14 captures physiological motion/artifact (see figure S1).

3. Verifying the Path Model with Complete Cases.

We calculated the effects of each of the four paths tested with relation to Hypothesis 2 in the main manuscript, with only the complete cases included (i.e., participants with any missing data were excluded; n = 44 included). Effect through the complete path holds: estimated effect = 0.007, bootstrapped SE = 0.004, bootstrapped 95% CI [0.001, 0.018]. Alternate paths omitting developmental measures do not hold, just as is the case for the analysis presented in the main manuscript.

Figure S1. Processing pipeline.

The three stages of fMRI data processing organized vertically from the top down, depicted with data inputs to each stage underlined, and subsequent analytic procedures boxed.

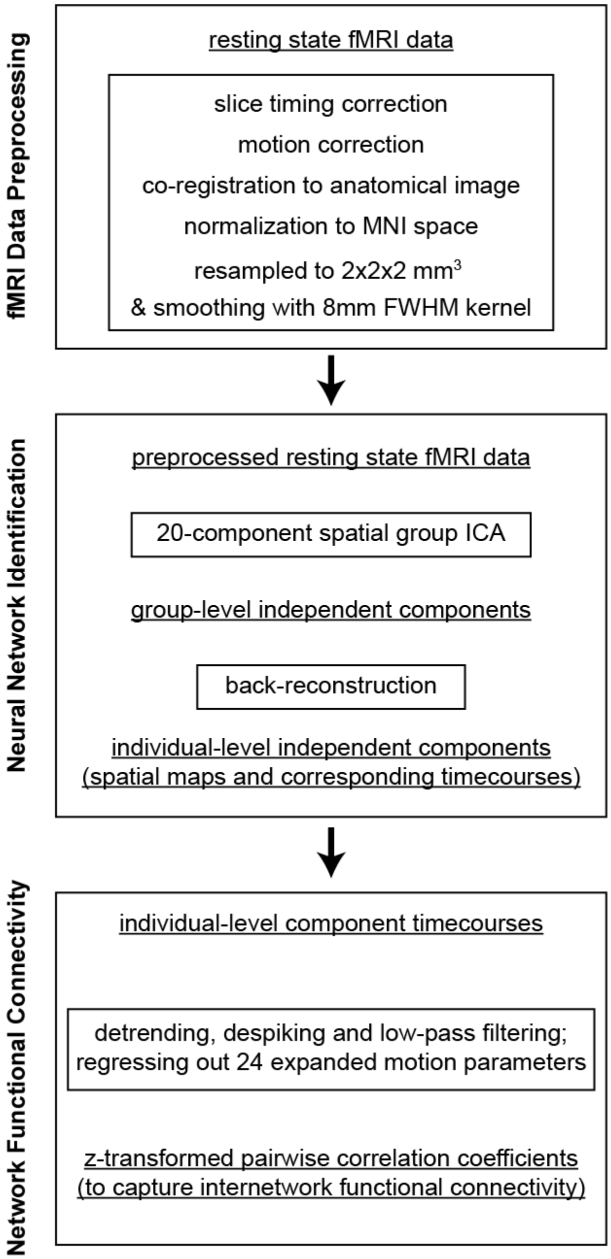

Figure S2. Time courses (A), power spectrum density plots (B), and spatial maps (C) for select group-level independent components from the 20-component spatial group ICA. Based on the spatial maps and the power spectrum profiles, component 11 captures the DMN; component 12 captures the left ECN; component 15 captures the right ECN; component 6 captures head motion; component 14 captures physiological motion/artifact.

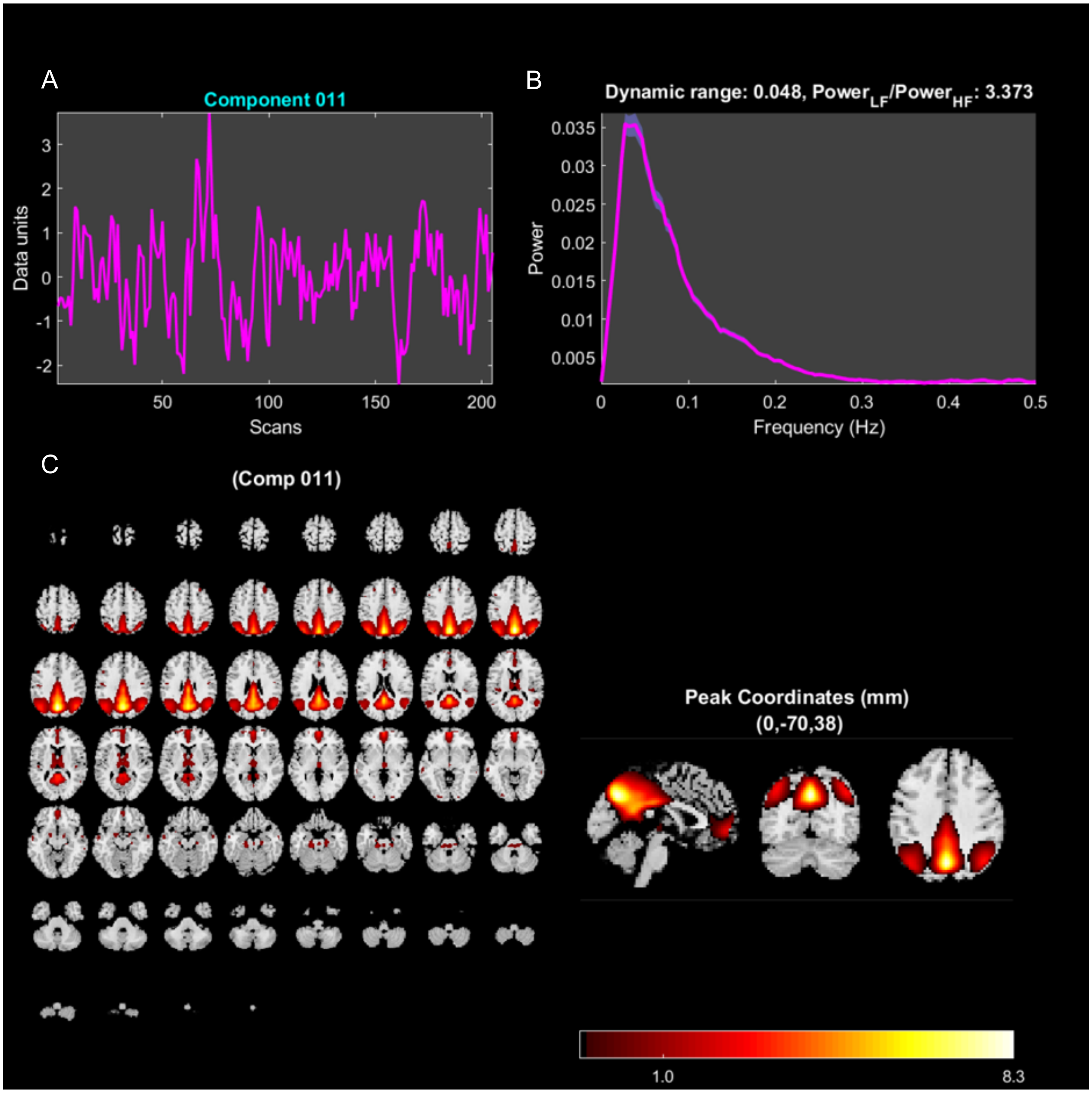

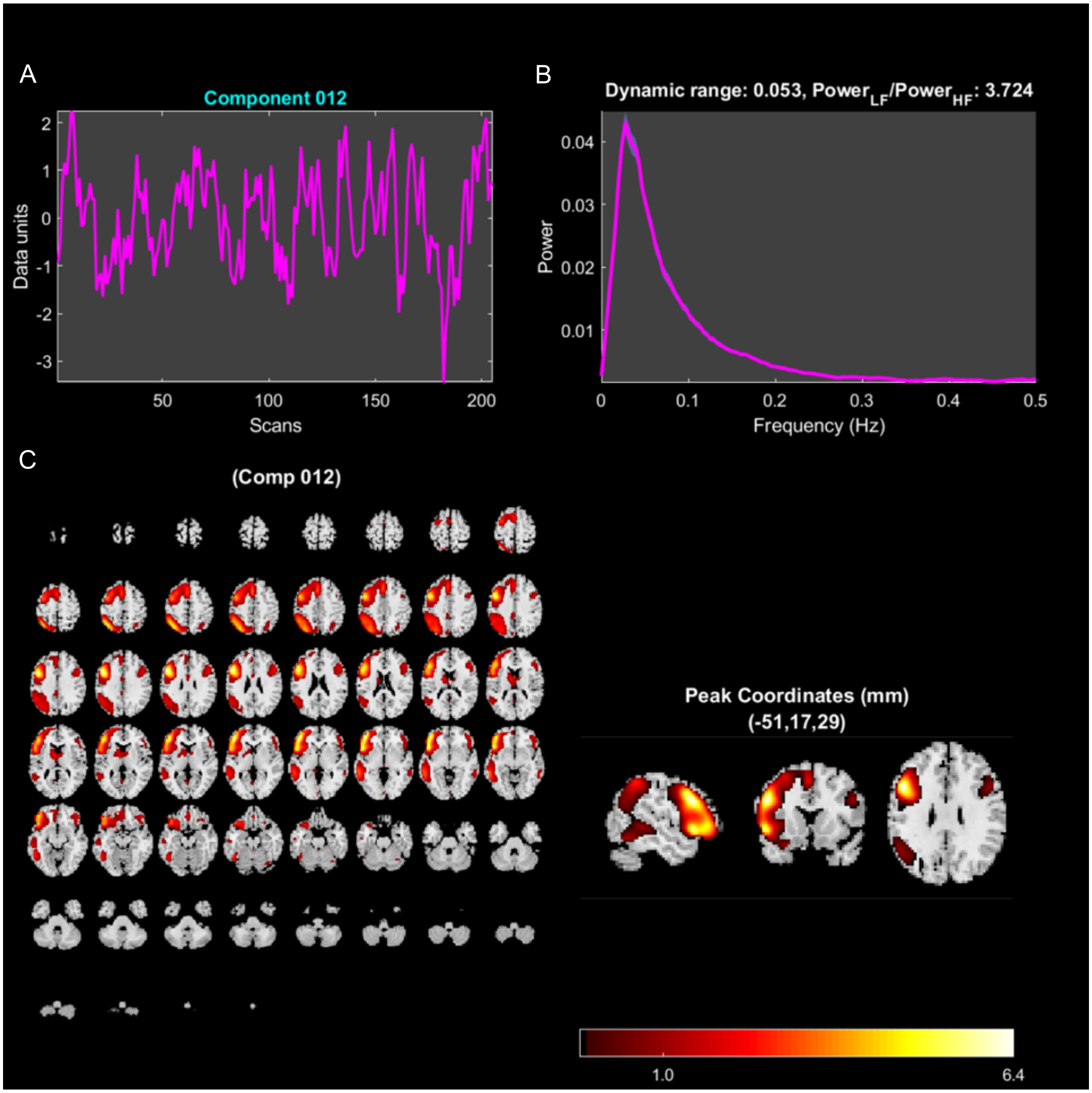
A
Component 012
Data units
Scans
B
Dynamic range: 0.053, Power_LF/Power_HF: 3.724
Power
Frequency (Hz)
C
(Comp 012)
Peak Coordinates (mm)
(-51,17,29)
1.0
6.4

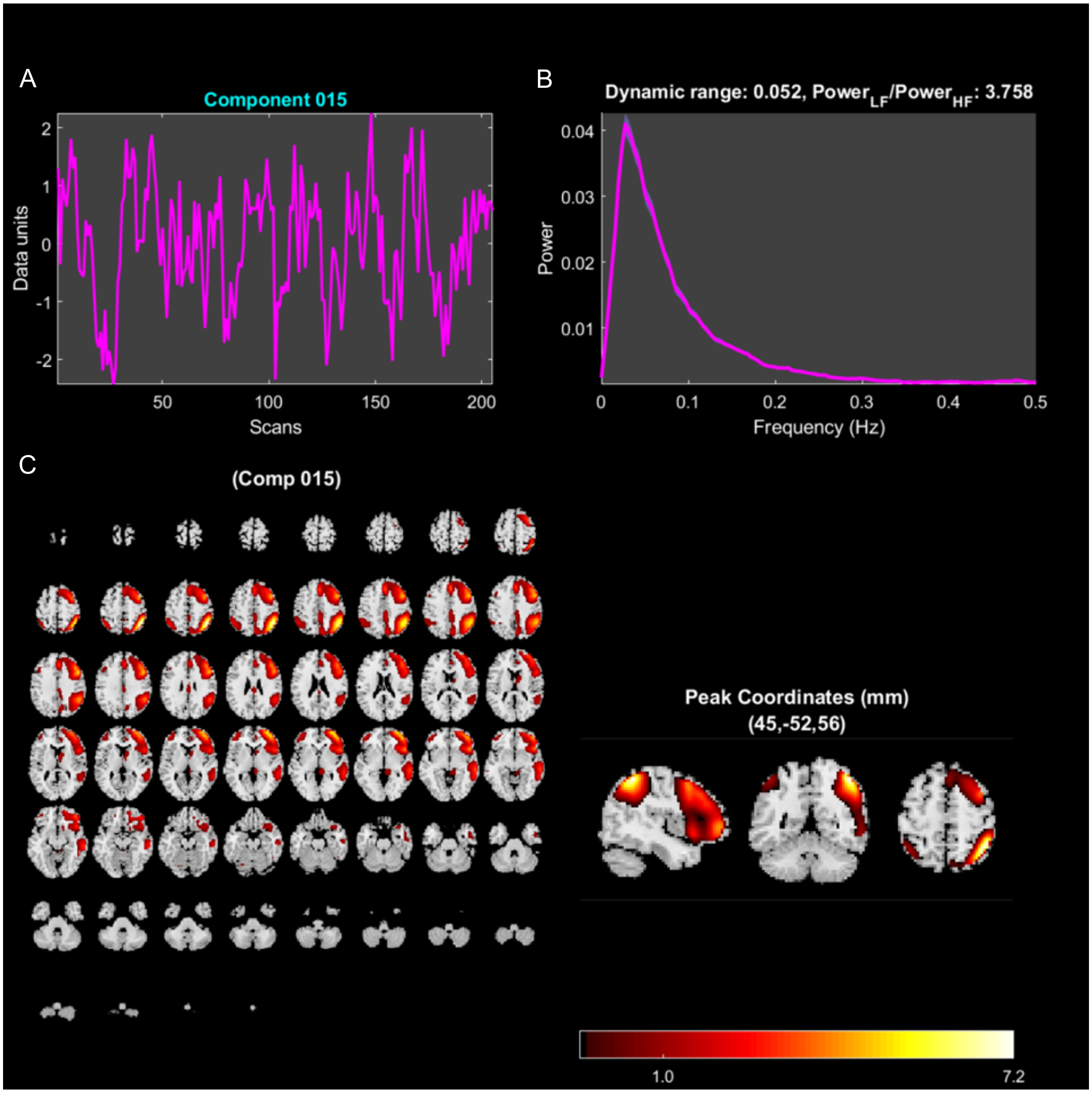

A
Component 015
Data units
Scans
B
Dynamic range: 0.052, Power$_{LF}$/Power$_{HF}$: 3.758
Power
Frequency (Hz)
C
(Comp 015)
Peak Coordinates (mm)
(45,-52,56)
1.0
7.2

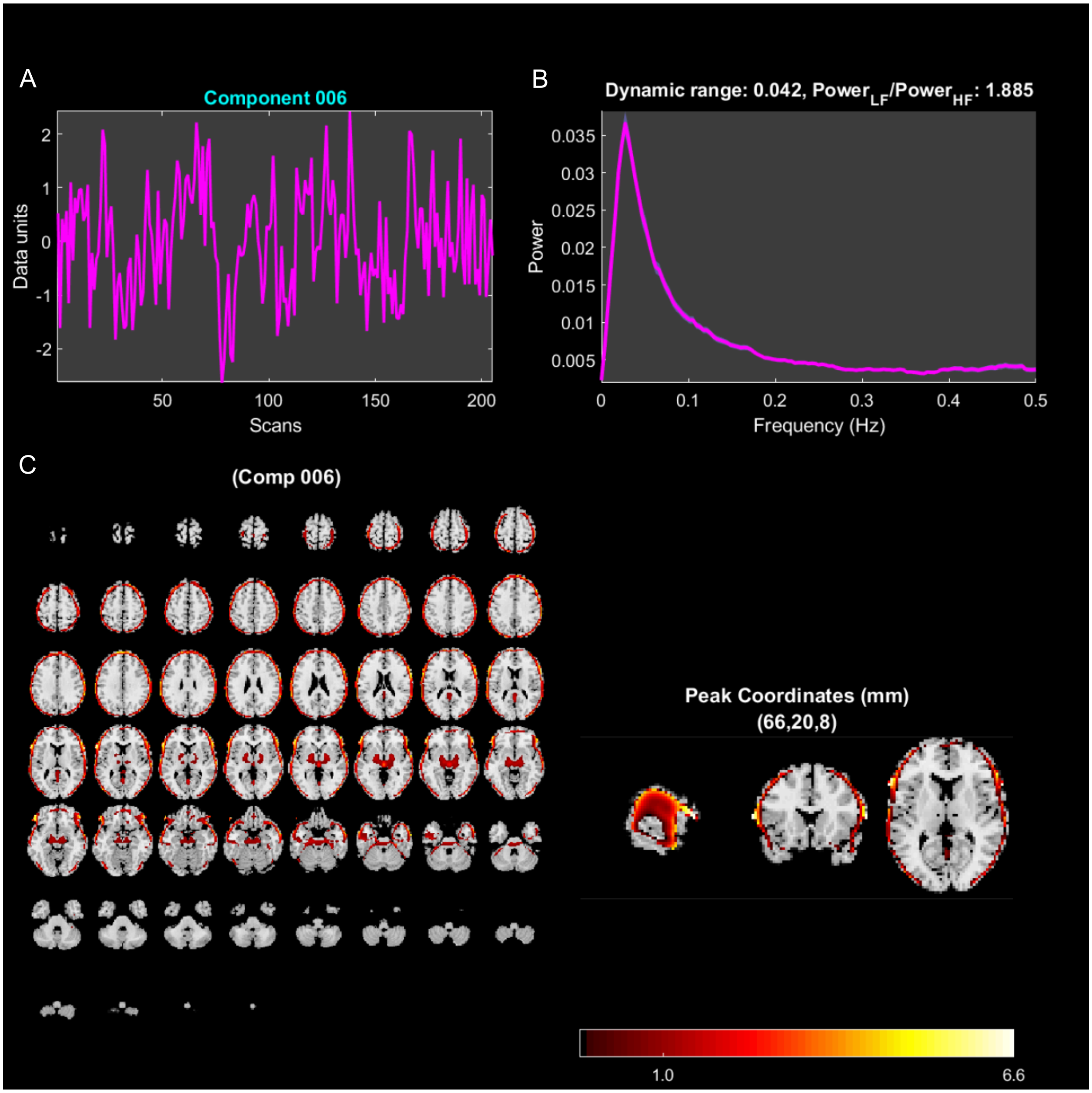
A
Component 006
Data units
Scans
B
Dynamic range: 0.042, Power_LF/Power_HF: 1.885
Power
Frequency (Hz)
C
(Comp 006)
Peak Coordinates (mm)
(66,20,8)
1.0
6.6

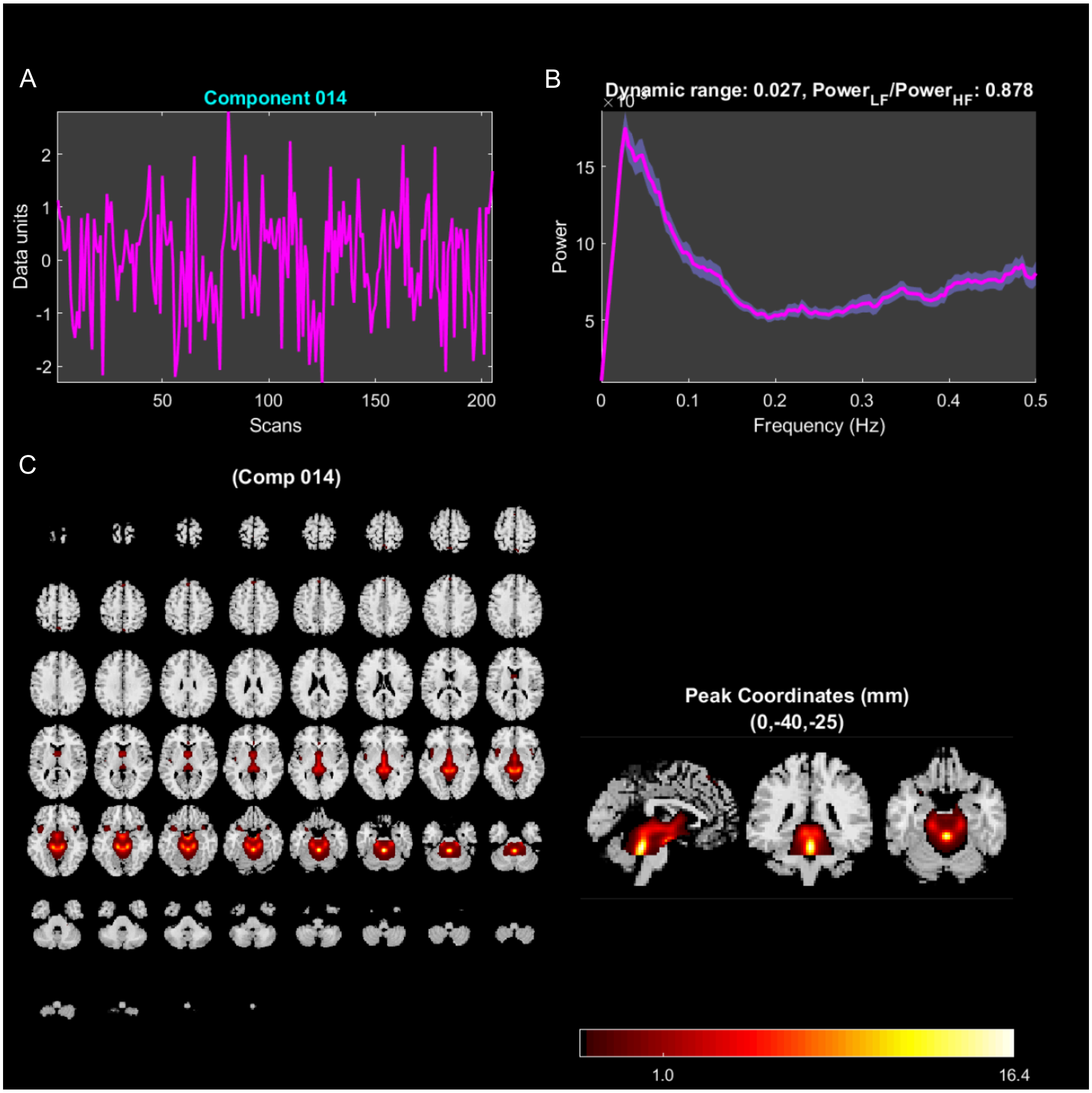
A
Component 014
Data units
Scans
B
Dynamic range: 0.027, Power_LF/Power_HF: 0.878
Power
Frequency (Hz)
C
(Comp 014)
Peak Coordinates (mm)
(0,-40,-25)
1.0
16.4

References

1. Frässle, S. *et al.* TAPAS: An open-source software package for translational neuromodeling and computational psychiatry. *Front Psychiatry* **12**, 680811 (2021).
2. Glover, G. H. H., Li, T. Q. Q. & Ress, D. Image-based method for retrospective correction of physiological motion effects in fMRI: RETROICOR. *Magn Reson Med* **44**, 162–167 (2000).
3. Smith, S. M. *et al.* Correspondence of the brain's functional architecture during activation and rest. *Proc Natl Acad Sci U S A* **106**, 13040–13045 (2009).
